# From High-Density Genomic Mapping to Precision Molecular Breeding: A Comprehensive Review of *Capsicum* Genomic Resources

**DOI:** 10.3390/genes17030298

**Published:** 2026-02-28

**Authors:** Luyao Wang, Junhu Kan, Weiting Zhong, Shuo Zhang, Yanghe Zhao, Yingke Hou, Luke R. Tembrock, Xiaolin Gu, Yan Cheng

**Affiliations:** 1 College of Horticulture, Shanxi Agricultural University, Taiyuan 030001, China; 2 Shenzhen Branch, Guangdong Laboratory of Lingnan Modern Agriculture, Key Laboratory of Synthetic Biology, Ministry of Agriculture and Rural Affairs, Agricultural Genomics Institute at Shenzhen, Chinese Academy of Agricultural Sciences, Shenzhen 518124, China; 3 School of Medical, Molecular and Forensic Sciences, Murdoch University, Murdoch, WA 6149, Australia; 4 College of Agriculture, National Beet Medium-Term Gene Bank, Heilongjiang University, Harbin 150080, China; 5 College of Plant Science & Technology, Huazhong Agricultural University, Wuhan 430070, China; 6 Department of Agricultural Biology, College of Agricultural Sciences, Colorado State University, Fort Collins, CO 80523, USA

**Keywords:** *Capsicum*, genomics, molecular breeding, cytoplasmic male sterility

## Abstract

The genus *Capsicum* comprises several species that are vital vegetable and spice crops cultivated worldwide, possessing significant economic, nutritional, and ornamental value due to their diverse fruit morphologies, colors, spiciness levels, and stress resistance. Historically, the large genome size (approximately 3 Gb) and high proportion of repetitive sequences (over 80% transposable elements) have constrained in-depth analysis of structural variations and functional genes within *Capsicum* species. However, recent advances in long-read sequencing, Hi-C scaffolding, and genome assembly have enabled the production of multiple high-quality and telomere-to-telomere (T2T) *Capsicum* genomes, which have ushered in a new era of research at the nuclear, organellar, and pan-genome levels. The publication of these omics resources has greatly expanded our understanding of the evolution of agronomically and environmentally relevant traits in peppers and their wild relatives. This review systematically summarizes recent progress in reference genomes, pan-genomes, and organellar genomes of the genus *Capsicum*, highlighting the enhancement of key breeding trait analyses through omics data, and outlines future integrated breeding strategies to provide theoretical and methodological references for genetic improvement and molecular breeding in pepper.

## 1. Introduction

The genus *Capsicum* (Solanaceae) originated in South America, with the first evidence of domestication dating back to about 6000 years before present from south central Mexico [1]. After colonization of the New World, *Capsicum* plants gradually spread to Africa, Asia, and other regions through human migration and trade. Pepper and chili pepper (for spicy cultivars), as the fruits of several *Capsicum* species are commonly known, are now one of the most widely cultivated vegetable crops in China and worldwide. The genus *Capsicum* currently includes over 40 species, comprising five domesticated species (*C. annuum*, *C. baccatum*, *C. chinense*, *C. frutescens*, and *C. pubescens*) and more than 30 wild species [2]. Peppers are regarded as a crop with important nutritional attributes as the fruits are known to be high in antioxidants and vitamins, while the spicy varieties are indispensable for adding “heat” to food through the presence of capsaicin. The rich diversity in fruit morphology, color, spiciness, and stress resistance makes *Capsicum* an ideal model lineage for studying plant secondary metabolism, domestication, and trait evolution through comparisons with extant wild relatives. Breeding and selection work in countries where peppers are an important crop has yielded a large number of cultivars with improved attributes as diverse as disease resistance to peppers such as the ghost pepper and the Carolina reaper that exceed one million Scoville heat units (a measure of pepper spiciness). However, with the expansion of omics resources and advanced algorithms, the advantages of targeted molecular design in breeding are becoming increasingly apparent, necessitating improved multidimensional development of underlying omics data and trait variations for the development of novel horticultural varieties [3,4].

The *Capsicum* genome is large and complex, approximately 3 Gb in size—three to four times that of tomato—with about 80% of the genome consisting of Long Terminal Repeat Retrotransposons (LTR-RTs) [5]. The high abundance of repetitive sequences led to fragmentation in early genome assemblies based on short-read sequencing, limiting the resolution of structural variations and annotation functional genes.

Prior to the widespread application of omics technologies, genetic research in pepper primarily relied on traditional segregating populations and Quantitative Trait Locus (QTL) mapping to identify loci associated with key agronomic traits. For instance, QTL mapping has been performed to investigate resistance to *Phytophthora* root rot [6]; Similarly, fruit shape and weight have been mapped to chromosomes 2 and 4, with the identification of the fruit shape QTL fs2.1 [7]; Additionally, yield-related traits were analyzed using recombinant inbred line (RIL) populations, leading to the discovery of multiple effect intervals [8]. Additionally, high-density maps and QTL analyses have been reported for traits related to the growth cycle and reproductive characteristics, such as the first flowering node [9]. Although these studies preliminarily revealed the genetic basis of certain traits, limited research tools and insufficient genomic data made it difficult to comprehensively elucidate the complex gene networks behind these traits and the genetic associations between species.

In the omics era, the discovery of pepper genes and traits has transitioned from “single-point breakthroughs” to “panoramic analysis.” With the application of third-generation long-read sequencing and high-throughput chromosome conformation capture (Hi-C) technologies, multiple high-quality, and even telomere-to-telomere (T2T) level, genomes have been constructed, laying a solid foundation for comparative genomics and functional gene mining in *Capsicum*. A 2025 study reconstructed the *Capsicum* ancestral karyotype and evolutionary trajectory using 11 high-quality genomes, identifying genome size variations driven by transposable element (TE) accumulation, 3D chromatin remodeling, and the evolution of fruit trait-related regulatory elements. The study also analyzed 124 core germplasm accessions, revealing that frequent interspecific introgression contributed to key traits in commercial varieties [10]. A plastome pan-genome (pan-plastome) aggregates chloroplast genome sequences from multiple individuals to capture both conserved and variable regions across accessions, providing a high-resolution framework for assessing plastid diversity, structural variation, and phylogenetic relationships. In *Capsicum* spp., such pan-plastome approaches have been applied to extensive sampling of cultivated accessions: for example, high-resolution plastome assemblies were generated from over 300 accessions representing multiple species complexes, enabling comprehensive comparison of plastome structure and sequence variation at the population level. Analyses of these pan-plastomes revealed both conserved gene content and polymorphic loci, including intergenic spacers and coding genes such as ccsA and other variable regions, that contribute to differentiation among *Capsicum* taxa and can be exploited as robust molecular markers for phylogenetic and taxonomic resolution [11]. Moreover, clustering based on plastome variation effectively groups accessions into clades that correspond with recognized species designations, illustrating the utility of the pan-plastome framework for germplasm classification and evolutionary inference in pepper. Furthermore, utilizing organelle genomics the mitochondrial genome of the cultivated species *C. pubescens* was completely sequenced for the first time [12]. The genome is approximately 454,165 bp in length and contains 35 Protein-Coding Genes (PCGs). Structural alignments and phylogenetic analysis revealed specific PCG loss/insertion, sequence translocation/inversion, and heterogeneity compared to other *Capsicum* mitochondrial genomes, providing critical data for exploring nuclear-cytoplasmic interactions and fertility mechanisms [13]. Pan-genomics analyses integrating cultivated and wild accessions, have revealed complex patterns of gene introgression and agronomic, challenging the traditional linear domestication model, while uncovering numerous gene families associated with environmental adaptability and domestication traits [14]. Developments in organelle genomics have further identified that mitochondrial genome rearrangements, chimeric gene formation, and nuclear-cytoplasmic interactions are key factors in the formation of Cytoplasmic Male Sterility (CMS) [15,16,17]. In summary, these omics-level studies have deepened research into the structural and functional evolution of the pepper genome and provided new theoretical support and technical methods for optimizing hybrid seed production systems and creating novel CMS materials.

This review systematically summarizes the major progress in *Capsicum* reference genomes, pan-genomes, and organellar genomes over the past decade, aiming to provide references for pepper genetic improvement and CMS research, as well as insights for future research directions.

## 2. The Evolution of *Capsicum* Reference Genomes

Research on the *Capsicum* genome is a history of scientific exploration driven by innovations in sequencing technology. From initially highly fragmented drafts to the complete T2T assemblies, each technological advance has further refined the complex genomic architecture and evolutionary patterns of pepper.

### 2.1. From Drafts to Telomere-to-Telomere (T2T) Assembly

**Figure 1:**
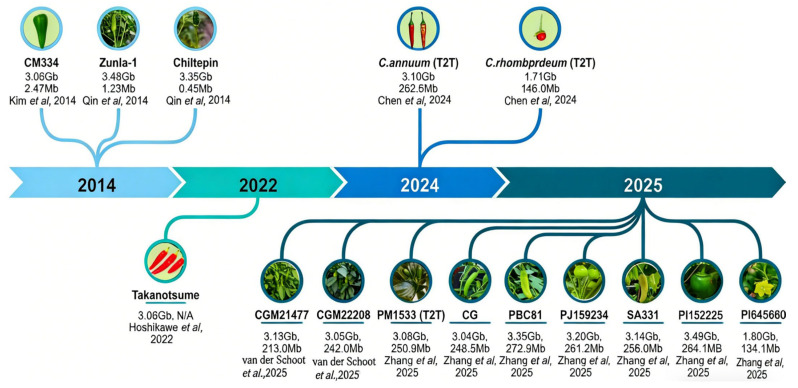
Timeline and key metrics of published *Capsicum* nuclear genome assemblies. Note: Data in this figure (from top to bottom) correspond to the cultivar name, assembly size, Contig N50, and reference(s), respectively. “N/A” indicates that the corresponding data were not explicitly reported in the original source materials [2,4,18,19,20].

The history of pepper genomics spans the leap from the short-read to the ultra-long-read era (Figure 1). Due to the inability of short-read technologies to span long repetitive regions in the genome, early genome drafts suffered from severe fragmentation and lack of continuity. For example, Kim et al. [18] first published a draft genome of the Mexican landrace *C. annuum* ‘CM334’ based on Illumina short-read sequencing, that generating approximately 650.2 Gb of raw data (approx. 186.6× coverage) but could only be assembled into 37,000+ scaffolds. Using SOAPdenovo for de novo assembly and SSPACE for scaffolding, the final assembly was approximately 3.06 Gb, representing 87.9% of the 3.48 Gb ‘CM334’ genome, and annotated about 35,000 coding genes. However, due to the abundance of repetitive sequences, the assembly was highly fragmented, consisting of 37,989 scaffolds with a scaffold N50 of only ~2.47 Mb, and many sequences could not be anchored to chromosomes. Similarly, the genome sequences of the cultivated *C. annuum* ‘Zunla-1’ and its wild progenitor ‘Chiltepin’ published by Qin et al. [5] were also based on Illumina sequencing, yielding assemblies of 3.48 Gb (Contig N50 ~1.23 Mb) and 3.35 Gb (Contig N50 ~0.45 Mb), respectively. Despite limited continuity, these assemblies have driven significant progress in pepper domestication research. For instance, comparison between ‘Zunla-1’ and ‘Chiltepin’ identified drought response LEA genes specific to ‘Chiltepin’ and the key capsaicin biosynthesis gene *Pun1* in ‘Zunla-1’, revealing for the first time the genetic basis for selecting spiciness during domestication.

To address the shortcomings of short-read technology, researchers gradually adopted PacBio and Oxford Nanopore long-read platforms, combined with optical mapping and Hi-C technologies, significantly improving assembly continuity and accuracy. Supported by these technologies, multiple high-quality chromosome-level genomes have been completed, including the Japanese cultivar ‘Takanotsume’, *C. chinense* [19], and a series of sweet peppers. Hoshikawa et al. [19] constructed a chromosome-scale assembly of the Japanese cultivar ‘Takanotsume’ (*C. annuum*) using PacBio HiFi reads, optical mapping, and genetic mapping. The assembly size was ~3.05 Gb, covering ~97% of the genome with a contig N50 of ~262.7 Mb. This study focused on dissecting gene clusters related to early fruit maturity and identified the MYB transcription factor family regulating intensity of spiciness, expanding the research perspective from single gene annotations to gene cluster functional associations.

Further improvements in sequencing precision and assembly algorithms led to the realization of T2T gapless genomes, signaling that genome research had entered the stage of “complete resolution.” In 2024, Chen et al. [20] published gapless T2T genomes for *C. annuum* and *C. rhomboideum* using a combination of PacBio long reads, Hi-C, Illumina, and ATAC-seq data. The genome sizes from this work were 3.10 Gb (Contig N50 ~262.6 Mb) and 1.71 Gb (Contig N50 ~146.0 Mb), respectively. The authors used ChIP-seq to precisely locate the centromeres of each chromosome. The study showed that, unlike many model species where centromeres rely on high-copy tandem repeats, pepper centromeres are not enriched in traditional repeats but are heavily invaded by retrotransposons, specifically CRM family elements. This study obtained the first gapless genome for the genus *Capsicum*, allowing for the mining of carotenoid synthesis genes controlling fruit color in *C. annuum* and specific insect protease inhibitor genes in *C. rhomboideum*. In 2025, Papastolopulou et al. [2] performed chromosome-level assembly for sweet pepper varieties CGM21477 and CGM22208, identifying loss-of-function mutations in the *Pun1* gene and expansion events in the sucrose transporter gene family related to fruit sweetness. Zhang et al. [10] published 11 high-quality genomes covering five cultivated and four wild species, with an average contig N50 exceeding 230 Mb, achieving T2T levels for *C. annuum* var. *glabriusculum* and *C. rhomboideum*. These studies have unveiled complex regions previously unresolvable, such as centromeres, telomeres, and rDNA clusters, providing high-resolution maps for exploring genome structure, evolution, and function.

### 2.2. Transposon Expansion Drives Genome Size Variation

**Figure 2:**
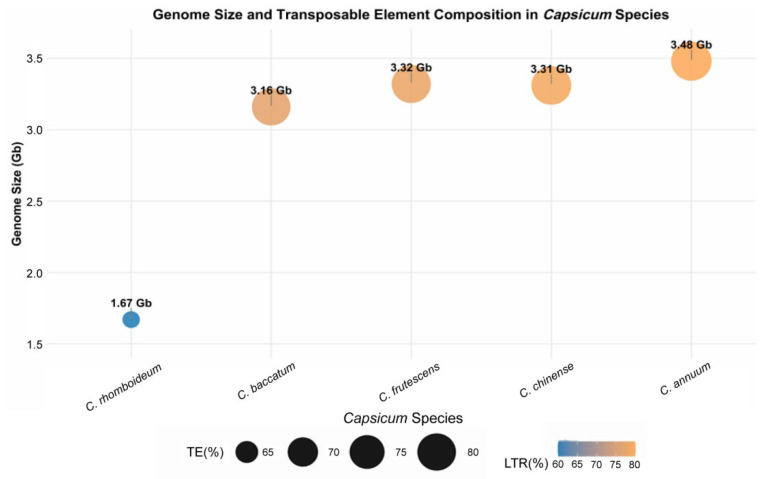
Comparative analysis of genome size, transposons, and LTR elements among different species in the genus *Capsicum*.

Prominent features of the *Capsicum* genomes sequenced thus far include relatively large sizes and significant size variation between species. Most pepper genomes are approximately 3.0–3.9 Gb, whereas the wild relative *C. rhomboideum* is only ~1.7 Gb (Figure 2). Studies indicate that this difference is not due to Whole Genome Duplication (WGD) events, as all *Capsicum* species retain traces of the same ancient WGD that occurred prior to the divergence of the genus. The *Capsicum* genome expansion appears to be driven primarily by TEs, particularly LTR-RTs. The timing and extent of TE expansions differ among species, leading to distinct differences in genome size and structure in the patterns of genomic divergence [21].

Approximately 80–85% of the pepper genome consists of TEs, with LTR-RTs being the core component. The LTR-RTs are mainly divided into the *Gypsy* and *Copia* superfamilies [2]. Within *Capsicum*, the *Gypsy-CRM* subfamily shows the most significant expansion, with copy numbers reaching millions in *C. annuum*, whereas this expansion is absent in *C. rhomboideum*. Genome size is significantly positively correlated with LTR-RT copy number; species with larger genomes like *C. annuum* and *C. chinense* experienced at least two explosive expansions of the *Gypsy* superfamily, while *C. rhomboideum* did not, resulting in its 1.7 Gb genome [21].

The TE insertion and expansion not only altered genome sizes but also played crucial roles in structural and functional evolution. TEs can remodel the 3D chromatin structure of genomes, affecting the expression of surrounding genes. For example, in *C. frutescens*, the insertion of a *Gypsy-Ogre* subfamily element in the upstream regulatory region of the fruit size regulator *FW2.2* introduced a new auxin response element (AuxRE), downregulating *FW2.2* and increasing fruit volume by approximately 40% [6]. TE insertions within genes or promoter regions can also create new regulatory elements. In *C. annuum*, a *Gypsy-CRM* element insertion in the promoter of the Capsaicin Synthase (*CS*) gene introduced a novel Jasmonate Response Element (JRE), enhancing *CS* response to jasmonate signals and increasing capsaicin accumulation [22]. Conversely, in sweet pepper (*C. annuum* var. *grossum*), a *Copia* element insertion in the coding region of *Pun1* caused premature termination of translation, resulting in the “non-spicy” phenotype [2].

### 2.3. Karyotype Evolution in Capsicum

Based on high-quality genomes, researchers have reconstructed the karyotype evolution in the genus *Capsicum*. Most pepper species have a chromosome number of 2n = 2x = 24 [2], while some species in the Andean clade (including *C. rhomboideum*) have 2n = 2x = 26.

Comparative genomic analysis reveals that the Ancestral *Capsicum* Karyotype (ACaK) consisted of 12 chromosomes [10]. The 13 chromosome pairs of *C. rhomboideum* formed through the fission and translocation of chromosome 12 of the ACaK [21]. This structural rearrangement left clear synteny break signals in the genome. Research also found that increases in chromosome number and genome size are not synchronous. For instance, *C. rhomboideum* has more chromosomes but a smaller, more compact genome with a lower proportion of LTR transposons. This suggests that chromosome number changes in *Capsicum* evolution were primarily driven by chromosomal fission/recombination, not solely by TE expansion.

**Figure 3:**
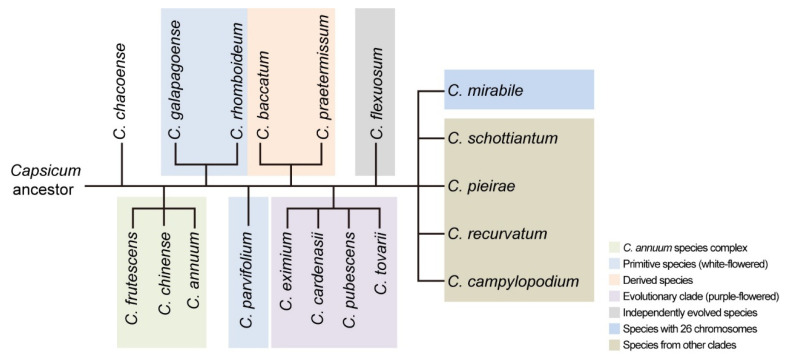
Evolutionary relationships of the genus *Capsicum* inferred from karyotype analysis (All *Capsicum* species share a common ancestor; the earliest derived species in the phylogenetic tree is *C. chacoense*, followed by the *C. annuum* species complex (*C. annuum*, *C. chinense*, and *C. frutescens*). Early derived species are white-flowered, including *C. galapagoense*, *C. rhomboideum*, and *C. parvifolium*. Derived species originated from *C. baccatum* and *C. praetermissum*. In addition, the genus *Capsicum* can be divided into three major evolutionary clades: (1) a clade of purple-flowered species comprising *C. eximium*, *C. cardenasii*, *C. pubescens*, and *C. tovarii*; (2) a lineage represented primarily by *C. flexuosum*; and (3) a group of species characterized by a basic chromosome number of 26, represented by *C. mirabile*. These results also reflect the complex and diverse patterns of cross-compatibility observed among different *Capsicum* species [23]).

Figure 3 illustrates the evolutionary relationships of the genus *Capsicum* inferred from karyotype analysis, clearly revealing the differentiation pathways of chromosomal structure and number among different species. Furthermore, this result indicates that the genomic evolution of *Capsicum* follows a “two-pronged approach”: one prong involves the rearrangement of chromosomal structure, and the other entails the gradual accumulation of transposons. These two processes are important in driving speciation through the formation of diversity at distinct levels.

## 3. Pan-Genomics Reveals the Landscape of *Capsicum* Genetic Diversity

To systematically analyze the rich genetic diversity of *Capsicum*, research has shifted from relying on single reference genomes to constructing representative pan-genomes.

### 3.1. Constructing the Capsicum Pan-Genome

A pan-genome represents a set of genomes that encompass all or nearly all genes within a given lineage such as a species or genus, and is typically divided into core, shell, and cloud gene sets. The core genome is made up of a set of genes and/or genomic regions that are shared by every genome in the set of sequenced genomes from a given lineage, the shell genome is made up of a set of genes and/or genomic regions that are shared by a majority sequenced genomes from a given lineage, and the cloud genome (sometimes also referred to as the peripheral genome or accessary genome) is made up of small subsets of genes and/or genomic regions shared by a small minority (or are unique to a single genome) of the sequenced genomes from a given lineage [24]. Pan-genomes are often categorized as open or closed using the degree to which core, shell, and cloud gene sets overlap with closed pan-genomes having large core genomes and small cloud genomes whereas open pan-genomes have small core genomes and large cloud genomes. Differences in pan-genomes are often associated with certain life history traits such as evolution to a particular niche, as in commensalist or parasitic lineages having closed genomes [25].

Pan-genome construction has facilitated the discovery of novel functional genes in pepper. In the first graph-based pan-genome built from 500 samples, including both wild and cultivated pepper accessions, 237 stress-resistance genes were identified in the cloud genome. An InDel variation in the promoter of the bacterial wilt resistance gene *PsyR1* explained the significantly higher resistance in wild materials compared to cultivars [26]. Additionally, a novel allele of the capsaicin synthase gene *CS* found in unique genes reduced capsaicin content by 40%, corresponding to a “low spiciness” trait [26]. Papastolopoulou et al. [2] integrated 16 high-quality genomes of the *C. annuum*, *C. baccatum*, and *C. pubescens* complexes, showing that only about 13% of genes are core genes, while 87% of variable genes are concentrated in the cloud genome. Such cloud genome genes are known to have agronomic importance, such as the NBS-LRR gene *Rps1*, which enhances resistance to *Phytophthora* blight by 80%.

In summary, studies on the *Capsicum* genome have revealed that core genes in the pepper genome are primarily responsible for fundamental life-sustaining processes such as basic metabolism and transcriptional regulation, functioning as housekeeping genes that sustain basic cellular processes [27]. In contrast, cloud genes are mostly involved in regulating traits related to environmental adaptation, including disease resistance, stress tolerance, and secondary metabolism [2]. This indicates that the greater the diversity of cloud genes, the better peppers can adapt to diverse environments and possess a wide range of traits—such as variations in disease resistance and flavor profiles.

### 3.2. Pan-Genomic Analysis of Domestication, Speciation, and Introgression

Studies analyzing whole-genome variations in hundreds of wild and cultivated accessions have elucidated the patterns of “wild-to-cultivated” directional selection [14,28]. For example, the fruit size regulating gene *CaFW2.2* is a core gene under strong selection; high expression in wild peppers inhibits cell proliferation, while promoter methylation in cultivars lowers expression, resulting in larger fruits. The *Pun1* gene variation marks sweet pepper domestication: wild peppers encode a functional enzyme, while cultivated sweet peppers carry a 2 bp frameshift mutation leading to function loss.

Pan-genomic comparisons have also revealed the role of gene introgression. The genome of modern cultivated pepper is a “mosaic” of multiple species. For example, large-fruited sweet peppers possess exogenous fragments conferring resistance to Pepper Mild Mottle Virus (PMMoV) [10].

Regarding reproductive traits, joint analysis of pan-genomes and expression profiles identified the “*orf138-Rf1*” module. The mitochondrial gene *orf138*, present only in certain *C. annuum* sterile lines, encodes a toxic protein disrupting pollen development. The fertility restorer gene *Rf1*, introgressed from *C. frutescens*, inhibits *orf138* expression to restore fertility [2,14,28]. These findings challenge the traditional “linear domestication model,” suggesting a “network model” where crop domestication involves frequent gene transfer and introgression among species.

With the continuous accumulation of high-quality genomes, research on the functional genes of *Capsicum* is gradually entering a “refinement phase”—the molecular mechanisms underlying multiple key traits such as spiciness, fruit morphology, and disease resistance are becoming increasingly clear [29,30,31]. Such understanding of genomic data also lays a solid genomic foundation for the subsequent in-depth exploration of the regulatory mechanisms governing male sterility and reproductive development.

## 4. Organelle Genomics and Its Application in Hybrid Breeding

Although smaller than nuclear genomes, chloroplasts (the most common plastid) and mitochondrial genomes play irreplaceable roles in plant metabolism, development and breeding, particularly in cytoplasmic inheritance and CMS.

### 4.1. Chloroplast Genome Dynamics and Phylogenetic Analysis

The *Capsicum* chloroplast genome is a typical circular double-stranded DNA structure with a conserved quadripartite structure [32], approximately 157 kb in length, and contains about 130 genes. Although chloroplast genomes are generally conserved, sequence variations such as insertions/deletions (InDels), single-nucleotide polymorphisms (SNPs), and simple sequence repeats (SSRs) are present among *Capsicum* species. These variations are primarily enriched in non-coding regions and at IR boundaries. For example, the *rps19* flanking sequence at the IRb/LSC boundary shows 20–50 bp InDel differences between *C. annuum* and *C. chinense*. These variations serve as effective molecular markers for phylogeny and germplasm identification.

In 2024, He et al. [33] sequenced and assembled the plastomes of 32 accessions from three *Capsicum* species, detecting hundreds of InDels and SNPs. Phylogenetic trees constructed from these data align with traditional taxonomy, clarifying the three main clades: the *C. annuum* complex, South American wild species, and the *C. pubescens* group [2]. In parallel, pan-plastome analyses of hundreds of cultivated *Capsicum* accessions have provided high-resolution assessment of plastid diversity and structure, resolving taxonomic boundaries among complexes and identifying polymorphic loci useful for phylogenetic and barcode analyses by integrating multiple accessions per species [11].

In addition, since chloroplast genomes exhibit maternal inheritance, the domestication history of the genus has been gradually elucidated by leveraging the differences in chloroplast genomes among different species or cultivars within the genus.

### 4.2. Mitochondrial Genome Structural Features and Recombination Mechanisms

#### 4.2.1. Structural Features

Plant mitochondrial genomes are characterized by large genome sizes, abundant repeats, frequent recombination, and extensive structural rearrangements. Despite structural plasticity protein-coding genes in mitochondrial genomes are relatively conserved among different species, whereas gene order and genome conformation frequently undergo changes [34,35]. These structural and sequence-level diversities provide a molecular basis for functional innovation, nuclear–cytoplasmic interactions, and adaptive evolution of mitogenomes.

In the genus *Capsicum*, the typical characteristics of plant mitogenomic diversity and recombination have been documented. Several whole mitogenomes have been completed in *Capsicum* with examples including Mahmoud et al. [36] that resolved the *Capsicum annuum* var. *glabriusculum* mitochondrial genome at a length of 505,190 bp, with 218 annotated genes (including 31 CDS, 3 rRNAs, and 25 tRNAs). Seona et al. [37] completed the mitochondrial genome from cultivated line *C. annuum* cv. ‘*Dempsey*’ at 577,453 bp in length, annotating 36 protein-coding genes (PCGs), 3 rRNAs, and 43 tRNA genes. Li et al. [13] completed the mitochondrial genome of *Capsicum pubescens* at 454,165 bp in length, with 35 PCGs, 30 tRNAs, and 3 rRNAs annotated, and the genome was found to have a multi-circular conformation. Comparative analyses have revealed significant differences in the order, orientation, and connectivity of gene blocks among the mitogenomes of different *Capsicum* cultivars, indicating the occurrence of rearrangement and recombination events. When using *C. pubescens* as a reference, alignments with the mitogenomes of other *Capsicum* cultivars have demonstrated large-scale structural variations, including block recombination and inversion of orientation. The structural plasticity of plant mitogenomes is further supported by evidence from other taxa [38]. Comparative analysis of mitogenomes from four Dendrobium species of *Orchidaceae* revealed a striking 1.6-fold variation in genome size ranging from 485 kb to 773 kb, coupled with complex genomic architectures characterized by the coexistence of circular and linear chromosomes. These mitogenomes also exhibit extensive repeat-driven structural rearrangements, alongside frequent gene gain and loss events—patterns that align closely with the structural diversity observed in *Capsicum* mitogenomes. This cross-family conservation reinforces that “structural rearrangement and repeat sequence accumulation” represent conserved evolutionary mechanisms shaping plant mitogenomes, offering valuable insights into deciphering the evolutionary drivers underlying gene block rearrangements in *Capsicum.*

Therefore, it is inappropriate to regard mitogenomes as static “conserved structures.” For the genus *Capsicum*, the mitogenomes not only exhibit variations in size but also show high plasticity and dynamism in terms of structure, repeat sequences, and gene block arrangement. Such diversity and variability provide a foundation for investigating interspecific differences, cytoplasmic inheritance, and associations with important traits (e.g., CMS).

#### 4.2.2. Recombination and Molecular Mechanisms of CMS

##### Progress in CMS Research

CMS is a pollen sterility phenomenon controlled by organellar genomes, typically caused by mutations or rearrangements of mitochondrial genes. Due to its maternal inheritance, understanding the mechanisms of CMS can improve hybrid seed production. CMS is usually induced by mitochondrial genomic mutations or rearrangements, which may lead to the emergence of CMS-associated *orf* genes in the mitogenome. Studies have shown that these CMS-related *orf* genes encode toxic transmembrane proteins, which can disrupt mitochondrial membrane structure or interfere with energy metabolism processes, resulting in the loss of male fertility in plants [39]. When the expression balance between nuclear and mitochondrial genes is disrupted, cellular energy supply and oxidative phosphorylation are impaired, ultimately causing abnormalities in stamen or pollen development [40].

**Figure 4:**
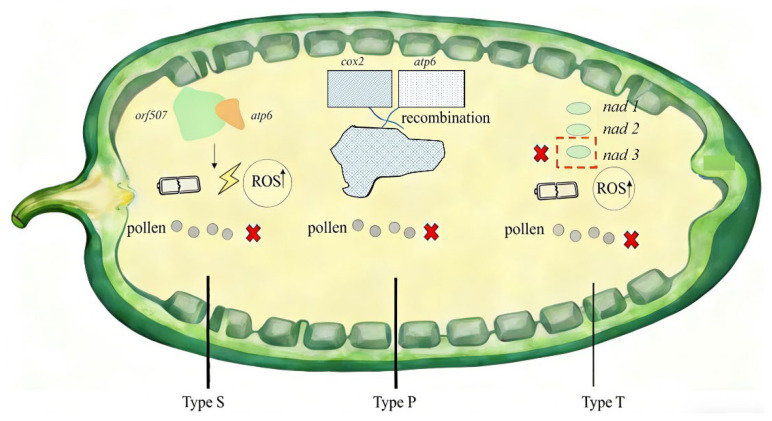
Schematic diagram of three typical CMS types in *Capsicum* (Type S): *orf507-atp6* interaction; (Type P): *cox2-atp6* recombination; (Type T): *nad* gene cluster deficiency.

To intuitively illustrate the diverse mitochondrial mechanisms underlying *Capsicum* CMS, three typical types (S, P, T) are summarized in a mitochondrial-localized schematic (Figure 4). Type S is triggered by the interaction of *orf507* and *atp6*: the abnormal association of their encoded proteins impairs ATP synthesis (reflected by the low-power battery icon) and elevates reactive oxygen species (ROS, marked by the lightning symbol), which jointly induce pollen abortion. For Type P, the recombination event between *cox2* and *atp6* (represented by the checkered chimeric structure) disrupts mitochondrial homeostasis, leading to the same sterile phenotype. Type T is linked to the dysfunction of the *nad* gene cluster (marked by the red cross on *nad3*): the impaired NADH dehydrogenase (a key component of respiratory chain) causes energy deficiency and ROS bursts, blocking pollen development. Despite distinct upstream genetic events, all three types converge on a core pathway.

Research on CMS in *Capsicum* dates back to the 1950s. Peterson [41] first identified the CMS phenomenon in the introduced accession PI164835 in 1958. Subsequently, Chinese researcher Yang Shizhou [42] isolated male sterile plants from the cultivar “Xiangyang Jiao” in 1984, successfully developed the sterile line 8021A and maintainer line 8021B, and established China’s first three-line matching seed production system for *Capsicum* [43].

In the era of molecular breeding, Kim et al. [44,45] identified *orf456* and the mitochondrial gene *atp6* as strong candidate genes for *Capsicum* CMS. Subsequent studies further discovered genes such as *orf507*, *orf300a*, and *orf314a*, all of which are closely associated with energy metabolism. Their abnormal expression may interfere with the normal function of cytochrome Coxidase and F_1_F_0_-ATP synthase, ultimately leading to pollen development abnormalities and sterility [27,46,47,48,49,50].

##### Development of Restorer Gene Markers

Restorer-of-fertility (*Rf*) genes are nuclear-encoded genes that restore fertility in CMS plants. With the advent of the molecular breeding era, researchers have utilized molecular marker-assisted selection (MAS) technology to conduct in-depth studies on the genetic basis of CMS and *Rf* genes. Zhang et al. [51] and Min et al. [52] developed RAPD markers tightly linked to *Rf* genes, providing early molecular tools for the selection of restorer lines. Subsequently, Lin et al. [53] and Gulyas et al. [54] established a high-precision SCAR marker (CRF-SCAR) for efficient screening of restorer lines. Lee et al. [55] further converted the polymorphic marker E-AGC/MGCA122 into a PR-CAPS marker, significantly improving the efficiency of *Rf* gene mapping and selection. Jo et al. [47,56] were the first to isolate the *Rf* gene from the nucleus and developed a co-dominant marker (Co1Mod1-CAPS) co-segregating with it, advancing the research on the molecular mechanism of *Capsicum* CMS from the genetic level to the functional validation stage.

With the development of high-throughput sequencing and comparative genomics technologies, molecular research on *Capsicum* CMS has entered the era of fine mapping. Min et al. [57] identified multiple AFLP markers linked to *Rf* genes by constructing a BSA (bulked segregant analysis) population. Wei et al. [58] established various molecular marker systems for rapid identification of *Capsicum* sterile lines by combining RAPD and SCAR technologies. Yang et al. [59] precisely mapped the *Rf* gene to chromosome 6 using SSR markers, laying a foundation for subsequent functional validation.

##### Nuclear-Cytoplasmic Interaction in CMS and Rf

Interactions between CMS and *Rf* genes have been extensively studied in other crops. For instance, BT-type CMS in rice is caused by the mitochondrial gene *orf79*, while the two restorer genes *Rf1a* and *Rf1b* encode pentatricopeptide repeat (PPR) proteins that specifically recognize and degrade abnormal transcripts, thereby restoring fertility [60]. WA-type CMS follows a similar mechanism: it is controlled by *orf352*, and fertility restoration also depends on *Rf1a/Rf1b* [61]. In maize, T-type CMS is induced by the mitochondrial gene *T-urf13*, but its restorer gene *Rf2a* does not belong to the PPR family; instead, it restores fertility by maintaining metabolic balance [62]. For pol-CMS in rapeseed, it is associated with the chimeric gene *orf224-atp6*, and the corresponding restorer gene *Rfp* encodes a PPR protein that achieves fertility restoration through processing or degrading abnormal transcripts [63]. Such mechanistic models indicate that nuclear genes functionally compensate for the abnormal expression of CMS-related mitochondria by regulating the processing, splicing, or degradation of abnormal mitochondrial RNAs, which represents the core mode of nuclear–cytoplasmic interaction. Recent studies have also made progress in understanding *Rf* genes in pepper as well. A recent study confirmed that the pepper restorer gene *CaRf* is a single gene encoding a mitochondrion-targeted PPR protein, which can significantly restore CMS fertility and has been applied in hybrid breeding lines. This demonstrates that pepper nuclear genes can also restore normal fertility through interactions with mitochondria [64].

Due to the high structural complexity of the CMS system in *Capsicum*, the mitogenome has undergone frequent recombination and segmental rearrangement during evolution, leading to the formation of various chimeric genes (e.g., *orf507*, *orf456*). The emergence of these chimeric structures results in significant differences among different sterile types, making *Capsicum* an important model for studying mitogenome remodeling and CMS formation mechanisms.

In summary, the formation of CMS in *Capsicum* is primarily driven by mitochondrial gene recombination. Newly generated chimeric genes often disrupt energy metabolism, leading to pollen abortion. In contrast, PPR-containing *Rf* genes act like “molecular scissors” that recognize and process these abnormal transcripts, thereby restoring pollen viability. With the increasing application of new technologies such as molecular markers, Genome-Wide Association Studies (GWAS), and mitoTALENs, the nuclear–cytoplasmic interaction mechanisms underlying *Capsicum* CMS should be comprehensively documented in the coming years. This will lay a solid molecular foundation for optimizing sterile systems and breeding more efficient hybrid varieties.

## 5. Future Perspectives: From Genomes to Integrated Breeding Strategies

### 5.1. Utilizing Multi-Omics to Enhance Trait Improvement

Future pepper research is progressively moving beyond a single-genome perspective and entering a new stage of multi-omics integration. Studies are expected to integrate data from genomics, transcriptomics, proteomics, and metabolomics to comprehensively dissect diverse agronomic traits in *Capsicum*, thereby providing strong support for molecular breeding.

For example, transcriptomics enables the quantitative profiling of dynamic gene expression across different tissues, developmental stages, or environmental conditions, offering direct evidence for elucidating fruit development, color formation, and capsaicinoid regulation. Metabolomics, in turn, can comprehensively analyze the accumulation patterns of various flavor, nutritional, and functional compounds in pepper fruits—such as sugars, organic acids, carotenoids, capsaicinoids, and flavonoids—thereby enhancing our understanding of the metabolic basis of important quality traits [65].

Moreover, by combining multi-omics data with genome-wide association studies (GWAS) and gene-metabolite network modeling, researchers can bridge the entire causal chain from “genetic variation to metabolic regulation to phenotype.” This integrated strategy enables researchers to more rapidly and accurately identify key genes controlling complex traits [66]. Indeed, numerous studies have successfully employed GWAS to identify candidate genes associated with fruit pigmentation, aroma, spiciness, and disease resistance [67,68,69,70], laying a solid foundation for marker-assisted selection (MAS) and targeted trait improvement in pepper.

In addition to genome-wide approaches, numerous functional gene-based markers have been developed and validated in *Capsicum* for trait improvement. These markers, derived from cloned genes or fine-mapped loci, provide direct tools for marker-assisted selection and precision breeding. Representative examples are summarized in Table 1.

### 5.2. The Critical Role of Accurate Whole-Plant Field Phenotyping in Capsicum Breeding

Despite the rapid expansion of genomic resources in *Capsicum*, including reference genomes, pan-genomics, GWAS, and gene editing platforms, the acquisition of accurate, high-resolution, whole-plant phenotypic data under field conditions remains a major bottleneck for precision breeding. Molecular data are only as informative as the phenotypes they explain or predict; without reliable, environment-aware phenotyping systems, genomics-assisted breeding cannot reach its full potential [78].

High-throughput phenotyping (HTP) technologies, such as UAV-based imaging, hyperspectral and thermal sensors, and automated image-analysis pipelines, now enable dynamic and large-scale trait quantification under field conditions [79,80]. Integration of high-quality phenotypic data with genomic analyses has significantly improved trait dissection and prediction accuracy in major crops [81]. However, compared with wheat, maize, and rice, systematic digital field phenotyping in *Capsicum* remains underdeveloped. Many key traits—such as yield stability, stress tolerance, and disease resistance—are strongly influenced by genotype–environment (G × E) interactions, which are difficult to capture through conventional manual assessments [82].

Future *Capsicum* research should prioritize standardized field phenotyping platforms, multi-environment trials, and robust G × E modeling frameworks integrated with GWAS, genomic selection, and machine learning approaches [83]. Ultimately, true precision breeding will require the tight integration of high-quality whole-plant phenotyping with genomic information to translate molecular discoveries into practical cultivar improvement.

### 5.3. Gene Editing Leading New Directions in Precision Breeding

With the rapid advancement of crop gene editing technologies, breakthroughs in gene editing and stable genetic transformation systems have become crucial prerequisites for achieving precision molecular breeding in *Capsicum*. Compared to model crops such as tomato, rice, and maize, the lag in in vitro regeneration and genetic transformation of *Capsicum* has long hindered the widespread application of gene editing technologies. Although studies have attempted various explant types and culture conditions to improve regeneration efficiency, truly efficient, stable, and reproducible *Capsicum* transformation systems remain scarce. Often relying on transient expression or protoplast editing rather than whole-plant transformation, this limitation has restricted the functional validation and promotion of gene editing in *Capsicum*. Wang Zhongyi from the team of Academician Zou Xuexiao [84] developed a *Capsicum* genetic transformation system by integrating a visual marker system and regeneration-promoting factors, which significantly enhanced adventitious bud regeneration efficiency and enabled CRISPR/Cas9-mediated gene editing with a 100% editing efficiency in T_0_ generation plants. This progress marks a significant step forward for *Capsicum* genetic transformation technology from the bottleneck of “unstable transformation” toward high efficiency and controllability, laying a foundation for subsequent precision breeding.

Building on this “editable + regenerable” platform, gene editing technologies such as CRISPR/Cas9 and CRISPR/Cas12a have gradually become core tools for *Capsicum* precision breeding. Compared to traditional hybridization or mutation induction methods, gene editing enables site-specific, directional, and quantitative precise modification at specific loci, greatly shortening the breeding cycle and improving modification efficiency. For example, CRISPR/Cas9-mediated editing of the disease resistance gene *CaMLO2* in *Capsicum* has achieved effective mutagenesis in leaf protoplasts of multiple cultivars, providing a molecular breeding tool for developing powdery mildew-resistant lines [85]. Similarly, CRISPR/Cas9 editing of the *CaPDS* gene involved in the carotenoid biosynthesis pathway has demonstrated distinct phenotypic changes, confirming the feasibility of efficient gene editing at the cellular level in *Capsicum* [86]. In addition, a DNA-free CRISPR/Cas9 delivery system based on viral vectors has achieved a heritable editing efficiency of up to 77.9% in *Capsicum*, offering a new approach to overcome the limitations of traditional transformation [85].

Notably, a recent review systematically summarizes the technical pathways and challenges of genome editing in polyploid crops [87]. For addressing key issues such as genomic redundancy and low editing efficiency of homologous genes in polyploids, precise modifications can be achieved through strategies including multi-guide RNA (gRNA) targeted design, base editing, and prime editing—approaches that have been validated in polyploid crops of Solanaceae and Poaceae. As a typical diploid crop (2n = 24/26), *Capsicum* does not face the constraint of genomic redundancy inherent to polyploids. Nevertheless, the insights proposed in this review, such as “optimization of precise editing tools” and “regulation of chromatin states to improve targeting efficiency”, provide valuable references for the efficient editing of genes associated with disease resistance and quality traits in *Capsicum*.

In other major food crops, improved genetic transformation systems and gene editing platforms have become important for precision breeding, providing a reference framework of ideal breeding models for recalcitrant crops such as *Capsicum*. The concept of “ideotype” was first proposed in 1968, referring to an ideal morphological model of plants expected to exhibit optimal yield or performance under specific environmental and cultivation systems [88]. This concept has since been widely applied in crop breeding design and optimization (i.e., crop “model plant architecture”). In soybean breeding, the CRISPR/Cas system has been used to precisely regulate plant architecture-related genes to modify architectural traits such as plant height, internode length, branching, and leaf type, thereby optimizing the three-dimensional structure of plants to enhance yield potential and water use efficiency. These achievements highlight the great potential of the gene editing-phenotype regulation system in optimizing plant architecture and yield [89]. In rice, to achieve high yield and cultivation adaptability, researchers have constructed ideal plant architectures based on targets such as dwarfism, erect leaves, and panicle type, and created allelic variations with the potential to improve single-plant yield by editing relevant regulatory loci. This provides a specific genetic basis and breeding strategy for the high-yield food crops [90]. For gramineous crops such as wheat, ideal plant architecture design also emphasizes optimizing the combination of multi-dimensional traits such as canopy structure, stress tolerance, and growth period, which helps achieve stable and high yields under different environments [91].

In recent years, with the integration of big data, biotechnology, and artificial intelligence (AI), crop breeding is entering a new era known as Breeding 5.0 or the smart breeding era. This concept emphasizes breaking away from traditional experience-driven and single-technical-path approaches and realizes “smart cultivation of smart varieties” by organically integrating genome editing, information technology, phenotypic big data, machine learning, and systematic breeding platforms. In other words, the bred varieties can better adapt to environmental changes, while the breeding process becomes more precise, efficient, and predictable. Academician Li Jiayang pointed out that Breeding 5.0 is no longer limited to molecular design breeding but integrates biotechnology and information technology to construct a smart variety framework capable of dynamically regulating superior traits, while improving comprehensive goals such as resource use efficiency, stress tolerance, and production performance [4]. Against this background, the technical route of *Capsicum* precision breeding should also shift from single gene editing optimization to a more systematic smart breeding system. Smart breeding not only emphasizes precise editing of key genes but also utilizes large-scale multi-omics data, AI-driven phenotypic prediction, and integrated analysis of multi-source data to achieve prediction and design of target traits. Constructing a smart breeding platform for *Capsicum* by closely integrating “stable genetic transformation + gene editing + multi-omics + AI-driven phenotypic prediction” is a key technical path to promote *Capsicum* from traditional molecular breeding to smart precision breeding.

In the field of molecular breeding, due to the rich phenotypic diversity and clear target traits in *Capsicum*, constructing a gene editing-based “ideal plant architecture” framework is crucial for precise improvement. As an important vegetable crop, the ideal plant architecture of *Capsicum* typically includes: a compact plant structure to facilitate high-density cultivation; a stable inflorescence structure and uniform fruit development to improve yield, controllable fruit shape and size to meet market demand; and disease and stress resistance to enhance cultivation stability and resistance levels. Meanwhile, different consumption types have specific preferences for flavor traits such as spiciness and fruit color, which are often determined by documented gene regulatory networks. The proposed ideal plant architecture of *Capsicum* not only emphasizes the optimization of basic agronomic traits but also considers quality and adaptability, providing a clear direction for the selection and functional validation of target sites for gene editing.

Therefore, constructing an efficient and universal *Capsicum* genetic transformation system is not only a prerequisite for the practical application of gene editing technologies but also an infrastructure for achieving *Capsicum* precision molecular breeding. Combining improved transformation and regeneration technologies, optimized gene editing platforms, and multi-omics association analysis between the nuclear genome and phenotypic traits, *Capsicum* is expected to develop into a model for precision breeding among non-model crops. This strategy integrating “stable genetic transformation + gene editing + systematic breeding analysis” can not only accelerate the improvement of key agronomic traits but also provide solid technical support for elucidating the molecular mechanisms of complex traits, realizing efficient hybrid breeding, and promoting smart germplasm innovation.

**Figure 5:**
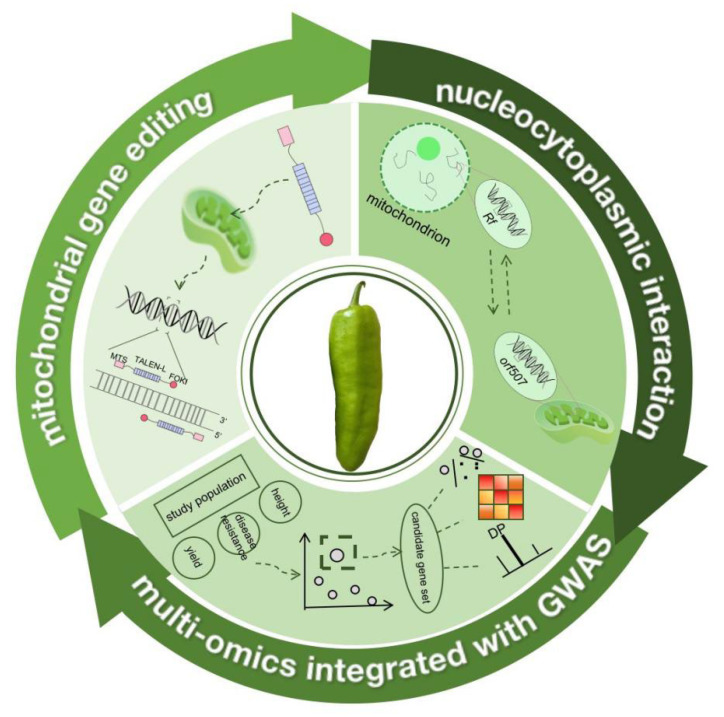
Perspective of *Capsicum* Molecular Breeding.

In the future, integrating the CMS system with gene editing technologies to achieve controllable construction of the sterility-restoration system by precisely editing mitochondrial genes and/or nuclear restorer genes (e.g., *Rf* genes) is expected to significantly improve the efficiency and flexibility of *Capsicum* hybrid seed production. It will also provide new research directions and theoretical support for clarifying the nuclear–cytoplasmic interaction mechanism and the molecular basis of cytoplasmic sterility (Figure 5). For mitochondrial gene editing, mitoTALEN technology is relatively mature, which can be used to edit CMS-related *orf* genes for functional validation and even construct CMS-restored lines. By integrating multi-dimensional data such as genomics, transcriptomics, and metabolomics with GWAS, key genes and regulatory networks affecting physiology, traits, and fertility can be systematically explored and validated. The three directions of mitochondrial gene editing, nuclear–cytoplasmic interaction, and multi-omics + GWAS form a closely coupled and synergistically advancing system of “smart breeding–mechanism elucidation–fertility control.” This research approach combining “nuclear–cytoplasmic interaction regulation” and “gene editing” will open up new directions for precision breeding of *Capsicum* and other Solanaceae crops in the near future.

### 5.4. Conclusions

Over the past decade, *Capsicum* genome research has evolved from “finding genes” to “using genes.” With the accumulation of T2T genomes and pan-genomes, combined with multi-omics and gene editing, pepper breeding is entering an era of intelligent predictive design. This integrated strategy will accelerate variety improvement and contribute to global food security.

## Figures and Tables

**Table 1:** Functional gene-based molecular markers reported in *Capsicum* and their applications in breeding.

Gene/Locus	Encoded Protein/Function	Trait	Marker Type	Molecular Basis of Polymorphism	Validation Approach	Breeding Utility	Reference
Pun1 (AT3)	Putative acyltransferase	Capsaicinoid biosynthesis (pungency)	SNP/Indel	Functional deletion in pungent vs. non-pungent genotypes	Gene cloning & co-segregation analysis	MAS for pungency control	Stewart et al., 2005 [71]
Pun3	Capsaicinoid pathway regulator	Non-pungency	SNP	Fine-mapped QTL region controlling capsaicinoid synthesis	Fine mapping & association analysis	Precision selection of mild cultivars	Zhu et al., 2019 [72]
CaMYB31	R2R3-MYB transcription factor	Capsaicinoid regulation	SNP	Expression polymorphism affecting structural genes	Expression profiling & functional validation	Regulation of pungency intensity	Arce-Rodríguez & Ochoa-Alejo, 2017 [73]
CaPSY1	Phytoene synthase	Fruit color (carotenoid biosynthesis)	SNP	Allelic variation influencing carotenoid accumulation	Sequencing & expression analysis	Fruit color improvement	Rodriguez-Uribe et al., 2012 [74]
CCS (L locus)	Capsanthin-capsorubin synthase	Red fruit pigmentation	CAPS/SNP	Functional mutation in carotenoid pathway gene	Genetic mapping & cloning	Red/yellow fruit selection	Popovsky & Paran, 2000 [75]
pvr2 (eIF4E)	Translation initiation factor	Potyvirus resistance	CAPS marker	Resistant allele conferring virus immunity	Allelism tests & mapping	Virus-resistant line development	Caranta et al., 1997 [76]
CaMLO2	Mildew resistance protein	Powdery mildew resistance	CRISPR-induced mutation	Loss-of-function mutation enhances resistance	Protoplast editing validation	Targeted disease resistance	Meng et al., 2019 [77]
Rf candidate gene	Restorer-of-fertility protein	CMS fertility restoration	SNP-linked marker	Nuclear restorer allele restoring pollen fertility	Linkage mapping	Hybrid seed production	Yeong Deuk et al., 2010 [47]

## Data Availability

No new data were created or analyzed in this study. All data discussed are from previously published articles, which are cited in the reference list.

## Abbreviations

The following abbreviations are used in this manuscript:

T2T	telomere-to-telomere
LTR-RTs	Long Terminal Repeat Retrotransposons
QTL	Quantitative Trait Locus
RIL	recombinant inbred line
TE	transposable element
PCGs	Protein-Coding Genes
CMS	Cytoplasmic Male Sterility
WGD	Whole Genome Duplication
AuxRE	auxin response element
CS	Capsaicin Synthase
JRE	Jasmonate Response Element
ACaK	Ancestral *Capsicum* Karyotype
PMMoV	Pepper Mild Mottle Virus
SNPs	single-nucleotide polymorphisms
SSRs	simple sequence repeats
ROS	reactive oxygen species
Rf	Restorer-of-fertility
MAS	marker-assisted selection
PPR	pentatricopeptide repeat
GWAS	Genome-Wide Association Studies
